# Diagnostic Performance of Quantitative Lung Perfusion SPECT/CT for Chronic Thromboembolic Pulmonary Hypertension: A Pilot Study

**DOI:** 10.3390/diagnostics16030413

**Published:** 2026-01-29

**Authors:** Yu-Sheng Liu, Yi-Ching Lin, Shih-Chuan Tsai, Hsin-Yi Wang, Jing-Uei Hou, Chia-Hung Kao

**Affiliations:** 1 Department of Nuclear Medicine, Taichung Veterans General Hospital, Taichung 407, Taiwan; yushengliu@vghtc.gov.tw (Y.-S.L.);; 2 Department of Medical Imaging and Radiological Sciences, Central Taiwan University of Science and Technology, Taichung 407, Taiwan; 3 Department of Post-Baccalaureate Medicine, College of Medicine, National Chung Hsing University, Taichung 407, Taiwan; 4 Department of Nuclear Medicine and PET Center, China Medical University Hospital, Taichung 407, Taiwan; 5 Artificial Intelligence and Robotics Innovation Center, China Medical University Hospital, Taichung 407, Taiwan; 6 Graduate Institute of Biomedical Sciences, College of Medicine, China Medical University, Taichung 407, Taiwan; 7 Department of Bioinformatics and Medical Engineering, Asia University, Taichung 407, Taiwan

**Keywords:** chronic thromboembolic pulmonary hypertension (CTEPH), lung perfusion, SPECT/CT, quantitative analysis

## Abstract

**Background:** Lung perfusion SPECT/CT is central to the diagnostic evaluation of chronic thromboembolic pulmonary hypertension (CTEPH), yet current assessments remain qualitative. This pilot study aimed to explore a standardized quantitative method for lung perfusion SPECT/CT to differentiate CTEPH from non-CTEPH patients. **Methods:** We retrospectively analyzed lung perfusion SPECT/CT studies obtained over a three-year period in patients assessed for suspected CTEPH. Perfusion counts were divided into ten equal intervals from zero to the maximum perfusion counts, and each decile was used as a threshold to define perfusion defects. Perfusion defect fraction was quantified, and group differences, diagnostic performance, and correlations with mean pulmonary arterial pressure (mPAP) were evaluated. **Results:** CTEPH patients showed significantly higher perfusion defect fraction than non-CTEPH controls. The 10% threshold demonstrated the best diagnostic performance, with an optimal cutoff of 20.6%, yielding a sensitivity of 75% and specificity of 100% for identifying CTEPH. Patients with distal-type disease or small, localized perfusion defects exhibited perfusion defect fraction overlapping with controls. Perfusion defect fraction correlated significantly and positively with mPAP. **Conclusions:** In this pilot study, quantitative analysis of lung perfusion SPECT/CT demonstrated feasibility as a complementary method to visual interpretation. While promising, these findings are preliminary and require validation in larger populations to establish their clinical utility for CTEPH diagnosis.

## 1. Introduction

Chronic thromboembolic pulmonary hypertension (CTEPH) is a subtype of pulmonary hypertension caused by pulmonary arterial obstruction from organized thromboembolic material and subsequent vascular remodeling [1]. Despite appropriate anticoagulation therapy, acute pulmonary embolism can progress to chronic macrovascular obstruction and microvascular disease. The resulting deterioration in pulmonary hemodynamics may ultimately lead to right ventricular failure and premature mortality. Unlike other forms of pulmonary hypertension, which are primarily managed with pharmacological therapy, CTEPH is potentially treatable through surgical pulmonary endarterectomy (PEA) or balloon pulmonary angioplasty (BPA) [2]. Consequently, current clinical guidelines suggest that CTEPH be excluded in all patients undergoing evaluation for pulmonary hypertension, using ventilation/perfusion (V/Q) lung scanning as the first-line imaging modality [3,4]. However, these recommendations presume the feasibility of ventilation imaging, which is frequently challenged in real-world clinical practice. Patients present with severe dyspnea, orthopnea, an inability to cooperate with the specific breathing maneuvers required for ventilation scanning, and communicable airborne diseases often preclude aerosol-generating ventilation procedures [5].

Consequently, lung perfusion-only single-photon emission computed tomography/computed tomography (SPECT/CT) has been adopted as a pragmatic alternative. Nevertheless, established diagnostic criteria or standardized quantitative frameworks specifically for perfusion-only SPECT/CT in the absence of ventilation data are currently lacking. Although SPECT/CT provides improved anatomical localization and image quality, interpretation in routine clinical practice remains largely qualitative or semi-quantitative [6,7,8,9]. Visual assessment alone cannot reliably quantify perfusion defect burden, assess treatment response, or allow standardized comparisons between CTEPH and non-CTEPH patients [10].

To bridge this gap between guideline recommendations and clinical reality, we hypothesize that quantitative analysis of lung perfusion SPECT/CT can differentiate CTEPH from other patient groups and provide a standardized framework for evaluating perfusion abnormalities.

## 2. Materials and Methods

### 2.1. Patients and Study Design

A single-center retrospective study was conducted to evaluate the feasibility of quantitative analysis for improving the diagnostic accuracy and standardization of lung perfusion SPECT/CT in patients with CTEPH. We retrospectively enrolled patients who underwent lung perfusion SPECT/CT at our hospital between March 2022 and March 2025 and had right heart catheterization (RHC) performed within a one-month interval. All patients underwent a comprehensive multimodality evaluation, including echocardiography and CT pulmonary angiography (CTPA). The diagnosis of CTEPH was confirmed by the following criteria: (1) pre-capillary pulmonary hypertension confirmed by RHC (mean pulmonary arterial pressure > 20 mmHg, pulmonary arterial wedge pressure ≤ 15 mmHg, and pulmonary vascular resistance > 2 Wood units); and (2) the presence of chronic thromboembolic obstructions confirmed by CTPA.

Patients were excluded if they had previously undergone pulmonary endarterectomy (PEA) or balloon pulmonary angioplasty (BPA); had a history of significant pulmonary disease; had a history of lung surgery; demonstrated poor image quality or image misregistration; had incomplete clinical or imaging data; or were pregnant at the time of examination. Patients diagnosed with CTEPH were assigned to the CTEPH group, whereas the remaining patients were included in the control group.

### 2.2. Image Acquisition

Lung perfusion SPECT/CT images were acquired immediately after the intravenous injection of Tc-99m MAA. A standard fixed dose was administered to all patients, with a mean administered activity of 161.5 ± 15.8 MBq. Images were acquired using either an Anger dual-head SPECT/CT gamma scanner (Discover NM/CT 670 Pro, GE HealthCare, IL, USA) or a 3D-ring general-purpose CZT SPECT/CT scanner (StarGuide, GE HealthCare, IL, USA) [11].

For the Discover NM/CT 670 Pro system, SPECT images were acquired using low-energy high-resolution collimators with a 128 × 128 matrix and 120 projections over 360°, with a dwell time of 10 s per view. Immediately following SPECT acquisition, a non-contrast, low-dose CT scan was performed without patient repositioning at 120 kV and 40 mAs for attenuation correction. SPECT images were reconstructed with both attenuation correction and scatter correction reconstruction.

For the StarGuide system, SPECT scans were acquired using 12 detector columns, each composed of seven modules containing 16  ×  16 CZT pixelated crystals with low-energy all-purpose collimators. A 512 × 512 matrix was used with variable detector motion. The acquisition included both a rotational motion along the gantry (2–6 steps per bed position with a rotation range up to 25°) and a sweep motion (±5–10° per step) [12]. Immediately following SPECT acquisition, a non-contrast, low-dose CT scan without patient repositioning was performed at 120 kV and 100–220 mAs for attenuation correction. Image reconstruction included both attenuation correction and scatter correction reconstruction.

All SPECT/CT images acquired from both imaging systems were reconstructed and analyzed using an Xeleris workstation (version 5.1, Q.Volumetrix AI; GE HealthCare, IL, USA), which allowed simultaneous review of SPECT, CT, and fused imaging datasets.

### 2.3. Quantitative Analysis of Images

Planar images were excluded as they lack the 3D data structure and CT-based attenuation correction required for the proposed volumetric quantification. Furthermore, the superposition of lung segments in 2D planar projections limits sensitivity compared to SPECT/CT [13]. The quantitative analysis was performed by an experienced nuclear medicine technologist and a physician who were blinded to the patients’ clinical information, RHC parameters, and final diagnostic grouping. Lung perfusion SPECT/CT images were analyzed using Xeleris workstation (version 5.1, Q.Volumetrix AI; GE HealthCare, IL, USA). The left and right lungs were segmented automatically using the Q.Volumetrix AI algorithm on the corresponding CT images, with the upper Hounsfield Unit (HU) threshold set at −400 by default to define the lung parenchyma. To ensure segmentation accuracy, a quality control procedure was applied. All automatically generated lung contours were visually inspected by an experienced nuclear medicine technologist and a physician. Manual corrections were performed slice-by-slice when necessary to adjust boundaries in cases of severe atelectasis or consolidation. The maximum perfusion counts were determined separately for each lung using post-reconstructed images, and the perfusion counts within each lung were divided into ten equal intervals ranging from zero to the lung-specific maximum count. For each perfusion count interval, the corresponding volume fraction was calculated by dividing the lung volume within the interval by the total lung volume. These volume fractions were subsequently used to compare perfusion distribution between the CTEPH and control groups.

**Figure 1:**
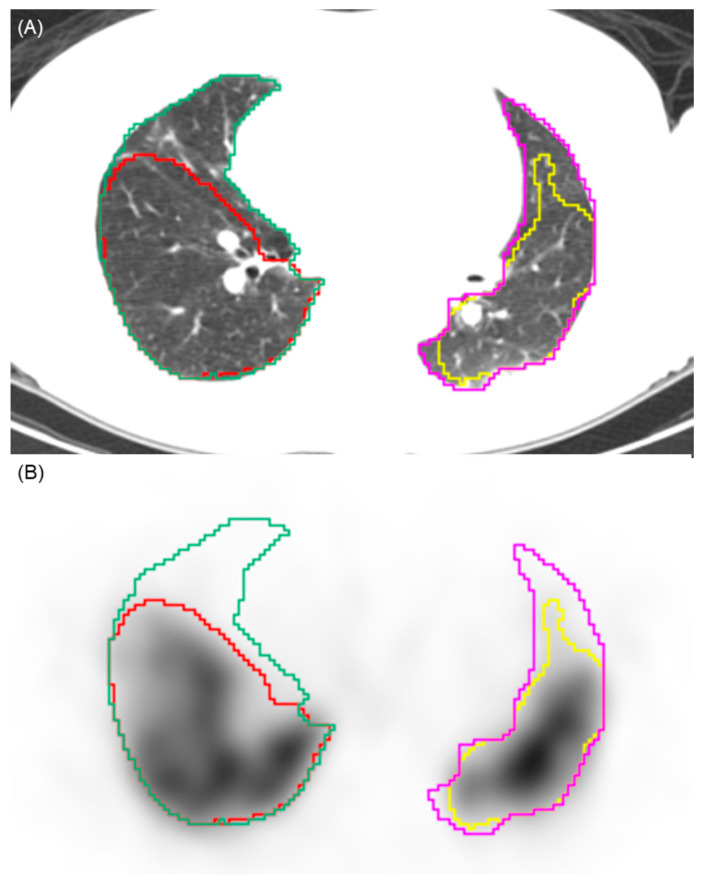
Illustration of the automated segmentation process. (**A**) Axial CT image showing the automated segmentation of the right (green contour) and left (pink contour) lung parenchyma. (**B**) Corresponding SPECT image demonstrating the application of a 10% perfusion count threshold. Voxels with counts above this threshold are segmented (yellow and red contours), while areas below this threshold are identified as perfusion defects. This visualizes the volumetric quantification methodology used to calculate the perfusion defect fraction.

To determine perfusion defects on SPECT/CT images, each decile value was applied as a threshold. Voxels with counts below the selected threshold were defined as perfusion defects, whereas those above the threshold were considered to represent normal perfusion (Figure 1). The perfusion defect fraction was then quantified under each threshold condition to assess its variation between groups and was calculated as the perfusion defect volumes of both lungs divided by the total lung volume of both lungs. The 10% threshold was pre-specified as the primary analysis parameter. This selection was based on the clinical practice for high sensitivity in screening for CTEPH [4]. Thresholds ranging from 20% to 90% were evaluated as an exploratory analysis.

### 2.4. Hemodynamic Data

Medical records were reviewed to obtain hemodynamic data, including systolic, diastolic, and mean pulmonary arterial pressures (sPAP, dPAP, and mPAP; mmHg), thermodilution-derived cardiac index (CI, L/min/m^2^), and pulmonary vascular resistance (PVR; Wood units). Peripheral oxygen saturation (SpO_2_) was measured at rest breathing room air.

### 2.5. Statistical Analysis

Continuous variables were evaluated for normality using the Shapiro–Wilk test. Variables with a normal distribution were expressed as mean ± standard deviation (SD) and compared using Student’s *t*-test, whereas non-normally distributed variables were presented as median (interquartile range, IQR) and compared using the Mann–Whitney U test. Categorical variables were compared using Fisher’s exact test. Receiver operating characteristic (ROC) curve analysis was performed to evaluate diagnostic performance. Confidence intervals (95% CIs) for sensitivity and specificity were calculated using the Clopper-Pearson exact method. The area under the ROC curve (AUC) was calculated, and internal validation was performed using bootstrap resampling (1000 iterations). The DeLong method was used to compare ROC curves. Spearman’s correlation was conducted to assess the associations between quantitative imaging parameters and mean pulmonary arterial pressure (mPAP). A two-tailed *p* value of < 0.05 was considered statistically significant. To account for multiple comparisons across the nine threshold levels (10–90%), a Bonferroni correction was applied, and a *p*-value of < 0.0056 (0.05/9) was considered significant for these specific comparisons. Statistical analyses were performed using MedCalc software (version 23.3.7; MedCalc Software Ltd., Ostend, Belgium).

## 3. Results

### 3.1. Patient Profiles

A total of 34 Asian patients were initially screened for this study, of whom 4 were excluded due to a diagnosis of chronic obstructive pulmonary disease. The remaining 30 patients were included in the final analysis, with 16 patients classified into the CTEPH group and 14 patients assigned to the control group. The baseline demographic and clinical characteristics of the participants are summarized in Table 1. No significant differences in age, sex, body height, body weight, lung volume, or hemodynamic parameters were observed between the control and CTEPH groups (all *p* > 0.05). However, peripheral oxygen saturation showed a borderline statistically significant trend (*p* = 0.0507), with lower values observed in the CTEPH group. The distribution of scanner systems is also presented in Table 1. No statistically significant difference in the proportion of scanner systems was observed between the CTEPH and control groups (*p* = 0.44).

### 3.2. Distribution Pattern of Lung Volume Fraction Between the CTEPH and Control Groups

**Figure 2:**
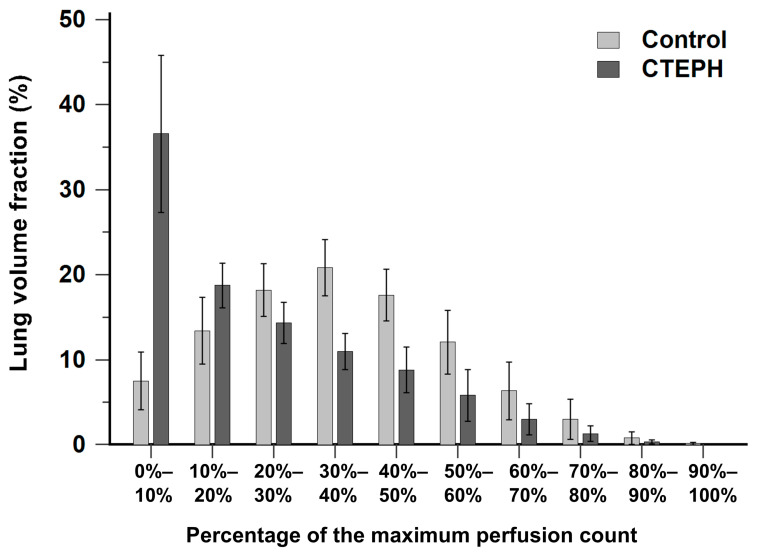
Bar chart showing the distribution of lung volume fraction across 10 perfusion-count intervals in the CTEPH and control groups. CTEPH indicates chronic thromboembolic pulmonary hypertension. Error bars indicate standard deviation.

Figure 2 illustrates the histogram of lung volume fraction across 10 perfusion-count intervals for both the CTEPH and control groups. The control group exhibited a distribution pattern approximating a normal distribution, with the majority of voxels concentrated within the mid-range interval of 30% to 40% of the maximal perfusion counts. In contrast, the CTEPH group demonstrated a distribution skewed toward low perfusion-count intervals, with a prominent peak occurring at the interval less than 10% of the maximal perfusion counts.

### 3.3. Comparison of Perfusion Defect Fraction Below Thresholds Between the CTEPH and Control Groups

**Figure 3:**
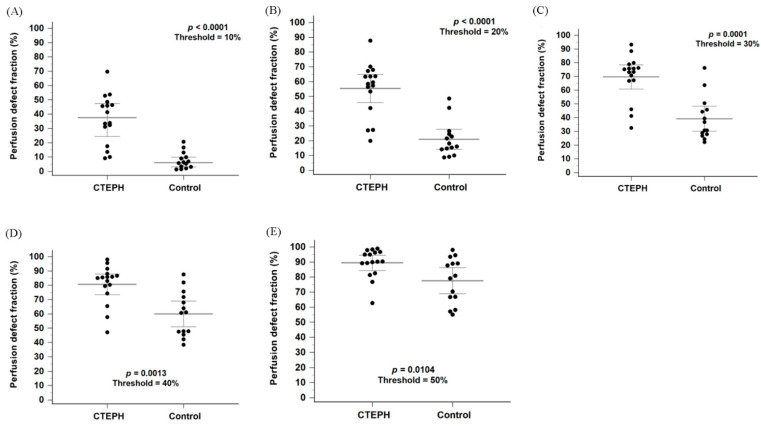
Box plots with scattered dots illustrating the perfusion defect fraction in the CTEPH and control groups using thresholds of 10%, 20%, 30%, 40% and 50% of the maximum perfusion counts, respectively (**A**–**E**). CTEPH indicates chronic thromboembolic pulmonary hypertension. Long horizontal lines denote median values, and short horizontal lines represent the interquartile range.

Figure 3A shows a box-and-whisker plot with scattered points of the perfusion defect fraction below 10% of the maximal perfusion counts for the CTEPH and control groups. The perfusion defect fraction in the CTEPH group was significantly higher than that in the control group (33.8% [16.6–46.9%] vs. 6.4% [2.8–10.7%], *p* < 0.0001). In addition, the perfusion defect fraction was significantly higher in the CTEPH group than in the control group at thresholds ranging from 10% to 40% (Figure 3B–D; all *p* < 0.0056). At the 50% threshold, the difference remained nominally significant (Figure 3E, *p* = 0.0104) but did not meet the Bonferroni-adjusted criterion. Within the heterogeneous control group, the Group 1 PAH subgroup (*n* = 10) exhibited a median defect fraction of 6.03% [3.40–13.2%] at the primary 10% threshold.

A subgroup analysis stratified by scanner systems was performed to evaluate possible confounding factors. The perfusion defect fraction remained significantly higher in the CTEPH group compared to the control group across both scanner systems at the thresholds between 10% and 30% (Supplementary Figure S1A–C; all *p* < 0.05). However, for the StarGuide system, the difference did not reach statistical significance at the 40% threshold (Supplementary Figure S1D, *p* = 0.07).

In Figure 3A, four patients in the CTEPH group showed perfusion defect fractions that could not be differentiated from those in the control group. While the diagnosis of CTEPH was confirmed by CTPA in all cases, further characterization via invasive pulmonary angiography revealed that these patients predominantly had distal-type CTEPH or small, limited perfusion defects. Detailed angiographic findings for these cases were summarized in Supplementary Table S1.

### 3.4. Sensitivity and Specificity of Perfusion Defect Fraction in Detecting CTEPH

**Figure 4:**
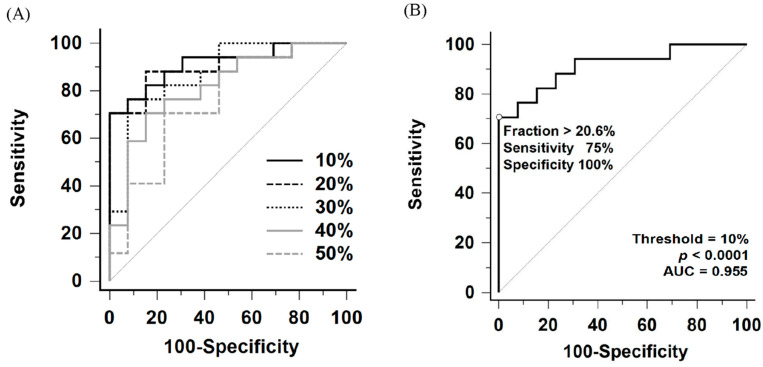
(**A**) Comparative receiver operating characteristic (ROC) curves of perfusion defect fraction for detecting CTEPH using thresholds ranging from 10% to 50% of the maximum perfusion counts. (**B**) ROC curve for detecting CTEPH using the 10% maximum perfusion counts threshold. AUC indicates area under the ROC curve.

Comparison of ROC curves for perfusion defect fraction across thresholds ranging from 10% to 50% of the maximal perfusion counts is shown in Figure 4A and Table 2. Among these thresholds, the 10% threshold yielded the largest area under the ROC curve (AUC = 0.955, 95% confidence interval [CI]: 0.811–0.997). Internal validation using bootstrap resampling (1000 iterations) yielded a 95% CI of 0.839–0.991. However, no statistically significant differences were observed when comparing the AUC of the 10% threshold with those of the 20% to 30% thresholds (10% vs. 20%, *p* = 0.57; vs. 30%, *p* = 0.18).

To evaluate the utility of this method as a screening tool, a lower cutoff of 9.1% under the 10% threshold was identified. This threshold was explicitly selected based on the ROC coordinates to maximize sensitivity. It yielded a sensitivity of 100% (95% CI: 79.4–100.0%) and a specificity of 71.4% (95% CI: 41.9–91.6%) (Table 3).

According to the Youden index, the optimal perfusion defect fraction cutoff was 20.6% under the 10% threshold, providing a sensitivity of 75% (95% CI: 47.6–92.7%) and a specificity of 100% (95% CI: 76.8–100.0%) for detecting CTEPH (Figure 4B and Table 3). Notably, all patients in the heterogeneous control group, including those with Group 1 PAH (*n* = 10), Group 2 PH (*n* = 3), and non-PH (*n* = 1), had perfusion defect fraction below the cutoff, resulting in 100% specificity across all subgroups.

A subgroup analysis stratified by scanner systems was performed to evaluate possible confounding factors. For the NM/CT 670 Pro system, the AUC at the 10% threshold was 0.958 (95% CI: 0.764–0.999). Using the screening cutoff (>9.1%), sensitivity was 100% (95% CI: 73.5–100.0%) and specificity was 62.5% (95% CI: 24.5–91.5%). Using the optimal cutoff (>20.6%), sensitivity was 83.3% (95% CI: 51.6–97.9%) and specificity was 100% (95% CI: 63.1–100.0%). For the StarGuide system, the AUC at the 10% threshold was 0.917 (95% CI: 0.575–0.999).

### 3.5. Correlation Between Perfusion Defect Fraction and mPAP in the CTEPH Patients

A significant positive correlation was observed between the perfusion defect fraction and mPAP in the CTEPH patients at lower thresholds. As shown in Table 4, Spearman’s correlation analysis revealed significant associations at the 10% (r_S_ = 0.61, *p* = 0.0129), 20% (r_S_ = 0.69, *p* = 0.0029), and 30% (r_S_ = 0.58, *p* = 0.0195) thresholds. However, the correlation did not reach statistical significance at higher thresholds (40% and 50%).

## 4. Discussion

In this pilot study, we demonstrated that quantitative analysis of lung perfusion SPECT/CT based on perfusion defect fraction may be feasible for distinguishing CTEPH from non-CTEPH patients and may provide a standardized method for evaluating patients with pulmonary hypertension.

Based on these findings, we propose a two-tiered clinical workflow using the 10% threshold: the 9.1% cutoff serves as a high-sensitivity ‘rule-out’ threshold to exclude CTEPH, whereas the 20.6% cutoff functions as a high-specificity ‘rule-in’ threshold to support the diagnosis. Several previous studies have explored quantitative methodologies for lung perfusion SPECT/CT in CTEPH [14,15,16]. Derlin et al. proposed a volumetric method to assess perfusion abnormalities and demonstrated correlations with clinical parameters in 30 patients with confirmed CTEPH, reporting a sensitivity of 88% and a specificity of 64% using a perfusion index with a 40% threshold [15]. However, their analysis was restricted to thresholds ranging from 15% to 85% of the maximal uptake. In contrast, our study focused on differentiating CTEPH from non-CTEPH patients and evaluated diagnostic performance of quantitative parameters across a broader range of thresholds, extending from 10% to 90%. The specificity reported in their study compared to our findings may be attributed to methodological differences and the specific thresholds selected. This discrepancy highlights that optimal threshold selection is dependent on the specific quantification technique and population, warranting further investigation. Similarly, Kuronuma et al. retrospectively investigated quantitation using the standard uptake value normalized to lung volume (SUV_LV_) in 58 patients, demonstrating patterns of lung volume distribution and diagnostic accuracy that are consistent with our findings, as illustrated in Figure 2 and Figure 4A,B [16].

In addition to establishing diagnostic accuracy, we also addressed the potential influence of equipment variability. A potential confounding factor in the present study was the use of two different SPECT/CT systems: a conventional Anger dual-head camera and a 3D-ring CZT camera. CZT detectors have different count statistics, spatial resolution, and reconstruction algorithms, which could theoretically influence quantitative metrics [12]. However, our subgroup analysis demonstrated that the diagnostic performance of the perfusion defect fraction was consistent across both scanner systems, particularly at the thresholds of 10% to 30% (Supplementary Figure S1). This finding supports the robustness of the proposed quantitative method, suggesting that the perfusion defect fraction at lower thresholds may maintain diagnostic validity across different scanner platforms. Nevertheless, further head-to-head studies comparing two different scanner systems to explore quantitative analysis of lung perfusion SPECT/CT may be needed to fully validate these findings.

We observed a moderate positive correlation between perfusion defect fraction and mean pulmonary arterial pressure. Consistent with our findings, both Derlin et al. and Kuronuma et al. reported significant correlations between perfusion defect extent and mean pulmonary arterial pressure, with correlation coefficients of 0.50 and 0.63, respectively [15,16]. Despite methodological differences among these studies, the concordant findings support the potential for standardized quantitative assessment using lung perfusion SPECT/CT. In contrast, Özgüven et al. retrospectively analyzed ventilation/perfusion SPECT/CT scans from 102 patients with CTEPH and reported a statistically significant but weak association (r = 0.09) between mean pulmonary arterial pressure and the number of lung segments with mismatched perfusion defects [17]. This discrepancy may be attributable to differences in quantification methodology, as their analysis was based on segment counts rather than volumetric or fractional assessment of perfusion defects. Beyond perfusion-derived metrics, echocardiographic indices of RV–pulmonary arterial coupling may provide complementary prognostic information in pulmonary hypertension. For instance, in systemic sclerosis–associated pulmonary hypertension, the TAPSE/sPAP ratio has shown predictive value for cardiovascular events and mortality, supporting the concept of integrated imaging–hemodynamic risk stratification [18]. Future studies combining our quantitative perfusion defect fraction with such echocardiographic parameters could further enhance risk assessment.

As shown in Figure 3A, four patients with distal-type CTEPH or CTEPH characterized by small, limited perfusion defects could not be distinguished from non-CTEPH patients based on perfusion defect fraction alone. Notably, all four cases were interpreted as positive for CTEPH on visual assessment by nuclear medicine physicians. While several quantitative imaging approaches have been proposed, visual interpretation continues to play a central role in CTEPH detection in current clinical practice and guidelines [3,4,19]. Tunariu et al. retrospectively evaluated ventilation/perfusion scintigraphy in 227 patients using the modified Prospective Investigation of Pulmonary Embolism Diagnosis (PIOPED) criteria, originally developed for acute pulmonary embolism, and reported a sensitivity of 96% and a specificity of 90% in detecting CTEPH [6,20]. In addition, a prospective study of 114 patients applying the modified PIOPED criteria for CTEPH diagnosis demonstrated a sensitivity of 100% and a specificity of 94% in ventilation/perfusion scintigraphy [7].

Furthermore, the European Association of Nuclear Medicine (EANM) proposed diagnostic criteria based on perfusion-ventilation mismatch involving at least one segment or two subsegments consistent with pulmonary vascular anatomy [19]. Wang et al. prospectively enrolled 208 patients and evaluated these criteria using ventilation/perfusion scintigraphy, ventilation/perfusion SPECT, and perfusion-only SPECT/CT. Although no significant differences in overall sensitivity or specificity were observed among the three imaging modalities, ventilation/perfusion SPECT demonstrated higher sensitivity, whereas ventilation/perfusion scintigraphy exhibited higher specificity for detecting individual segmental defects [8]. Conversely, a retrospective study by Sung et al. applying the EANM criteria reported significantly lower specificity for perfusion-only SPECT/CT in the diagnosis of CTEPH [9].

Dual-Energy CT (DECT) and subtraction CT iodine mapping have gained prominence for their ability to provide high-resolution, simultaneous assessment of pulmonary anatomy and perfusion [21]. However, the widespread clinical utility of these techniques is restricted by specific patient contraindications. The primary limitation lies in the reliance on iodinated contrast media, which renders DECT and subtraction CT unsuitable for patients with renal insufficiency. Similarly, patients with a history of severe contrast hypersensitivity or unmanaged thyroid dysfunction are precluded from these CT-based perfusion assessments. In contrast, lung perfusion SPECT/CT utilizes Tc-99m MAA, avoiding the nephrotoxicity and allergic risks associated with iodinated contrast. The quantitative method proposed in this study bridges the gap by enabling standard SPECT/CT to provide perfusion metrics without the associated contraindications.

Our analysis confirmed that specificity was maintained across the heterogeneous control group. While Group 1 PAH is characterized by mottled, non-segmental perfusion defects that are traditionally distinguished from CTEPH via expert visual interpretation, our findings imply that the total perfusion defect fraction in PAH may be significantly lower than in CTEPH [22,23]. Nevertheless, we acknowledge that the present study focused on volumetric quantitative analysis and did not incorporate morphological features such as the shape or anatomical distribution of perfusion defects. These features may be important for differentiating distal-type CTEPH or cases with small, limited perfusion defects from Group 1 PAH, and can be recognized by experienced nuclear medicine physicians during visual interpretation. Quantitative methods incorporating such characteristics, such as laterality or the fractal dimension proposed by Maruoka et al., have been explored [14]; however, their clinical utility requires further validation. Future studies incorporating texture analysis or shape features may further enhance diagnostic precision, particularly in borderline cases.

Distal-type CTEPH is characterized by hemodynamic features that differ from those of central-type disease and is generally considered more amenable to balloon pulmonary angioplasty (BPA) than to surgical pulmonary endarterectomy (PEA) [24,25,26,27]. A national cohort study of Korean patients with CTEPH reported that 21.6% of cases were classified as distal-type and demonstrated lower rates of PEA compared with central-type disease [28]. Similarly, a nationwide prospective registry in Japan reported a higher prevalence of distal-type CTEPH in Japanese populations, with BPA more frequently selected as the treatment modality, in contrast to Western cohorts in which PEA predominates [29,30]. In this context, our findings suggest that perfusion defect fraction alone may be insufficient for reliably identifying distal-type CTEPH or cases with minimal perfusion abnormalities, particularly in Asian populations. Additional or alternative quantitative imaging parameters should therefore be explored.

Based on these observations, we propose that the perfusion defect fraction should be integrated into clinical practice as a complementary tool alongside expert visual interpretation, rather than as a replacement. Furthermore, this standardized approach provides a baseline for disease severity, which is particularly valuable for monitoring treatment response.

CTEPH is potentially treatable through PEA or BPA, and several studies have explored quantitative imaging approaches for monitoring treatment response. Maruoka et al. demonstrated the utility of volumetric perfusion SPECT analysis using a fractal dimension threshold of 2.4 to assess post-BPA response [14]. Kuronuma et al. reported that standard uptake value normalized to lung volume was useful for detecting hemodynamic improvement after BPA [16]. Staal et al. retrospectively evaluated 33 patients with CTEPH and observed significant post-BPA improvement on ventilation/perfusion scintigraphy using visual and semiquantitative assessment [31]. Additionally, Han et al. retrospectively analyzed ventilation/perfusion SPECT images in 74 patients using volumetric methods and reported their ability to predict persistent or recurrent pulmonary hypertension after PEA [32]. Although the present study focused on CTEPH diagnosis, these findings highlight the broader potential of quantitative lung perfusion imaging for treatment monitoring, warranting further prospective investigation.

This study has several limitations. First, it was a single-center retrospective pilot study with a small sample size. This limited statistical power likely precluded the detection of significant differences in categorical clinical characteristics, such as WHO functional class, despite observed trends. Additionally, all participants were of Asian ethnicity, which may limit generalizability. Second, SPECT/CT data were acquired on two different scanner systems with vendor-specific reconstruction protocols, and no physical phantom cross-calibration was performed to harmonize absolute counts, which may introduce variability in image characteristics and quantification. Third, the maximum counts were defined as the peak voxel value within the segmented lung volume. Other techniques, such as using a smoothed or robust maximum as a quantification reference, may further reduce susceptibility to hotspots or noise and are worth investigating. Fourth, we did not perform a formal assessment of inter-observer or intra-observer variability. Although the quantitative analysis employed in this study primarily utilized automated lung segmentation and fixed perfusion thresholds to minimize subjective bias, the potential influence of manual adjustments to lung contours warrants further investigation. Fifth, the control group consisted of a heterogeneous mix of non-CTEPH patients, primarily including Group 1 PAH. In addition, our study excluded patients with significant parenchymal lung diseases. We acknowledge that this exclusion introduces spectrum bias, as these conditions are common causes of perfusion heterogeneity and may produce false-positive defects in a real-world clinical setting. Interpretation of quantitative perfusion defects in patients with significant parenchymal lung pathologies requires caution. Future studies with more homogeneous control groups and prospective, multicenter studies are warranted to validate these findings on lung perfusion SPECT/CT in CTEPH.

## 5. Conclusions

This pilot study suggests the feasibility of using quantitative analysis of lung perfusion SPECT/CT as a diagnostic tool for CTEPH. These preliminary results support the potential of this quantitative approach to serve as a complementary quantitative metric to visual interpretation, warranting further investigation in larger, multicenter studies.

## Figures and Tables

**Table 1:** Baseline characteristics of patients.

	CTEPH (*n* = 16)	Control (*n* = 14)	*p* Value
Age (years)	64.1 ± 13.2	56.6 ± 12.7	0.1261
Male sex, n (%)	6 (37.5)	5 (35.7)	1.0000
Body height (cm)	157.2 (152.5–164.3)	161.2 (152.3–165.2)	0.7552
Body weight (kg)	60.0 (53.8–70.2)	58.0 (48.0–70.2)	0.8538
Lung volume (mL)	2413 (2036–2774)	2157 (1933–2556)	0.5799
Heart rate (bpm)	77 (73–87)	78 (69–83)	0.8188
SpO_2_ (%)	95 (94–97)	97 (95–98)	0.0507
WHO functional class I/II/III/IV	1 (6)/10 (63)/5 (31)/0 (0)	1 (7)/12 (86)/1 (7)/0 (0)	0.2557
WHO clinical classification			<0.0001 *
Group I	0 (0)	10 (72)	
Group II	0 (0)	3 (21)	
Group IV	16 (100)	0 (0)	
Not PH	0 (0)	1 (7)	
sPAP (mmHg)	53 (37–74)	61 (42–77)	0.7708
dPAP (mmHg)	23 (17–30)	28 (20–38)	0.2703
mPAP (mmHg)	35 (28–43)	45 (32–53)	0.4052
PVR (WU)	6.1 (4.0–12.4)	8.7 (5.2–11.2)	0.4213
CI (L/min/m^2^)	2.5 (2.2–2.8)	2.3 (1.9–2.6)	0.3198
Scanner system			0.4421
NM/CT 670 Pro	12 (75)	8 (57)	
StarGuide	4 (25)	6 (43)	

Abbreviations: CTEPH, Chronic Thromboembolic Pulmonary Hypertension; SpO_2_, peripheral oxygen saturation; sPAP, systolic pulmonary arterial pressure; dPAP, diastolic pulmonary arterial pressure; mPAP, mean pulmonary arterial pressure; PVR, pulmonary vascular resistance; WU, Wood unit; CI, cardiac index. * indicates *p* < 0.05.

**Table 2:** Area under the ROC curve at different thresholds.

Threshold	AUC (95% Confidence Interval)
10%	0.955 (0.811–0.997)
20%	0.946 (0.798–0.996)
30%	0.897 (0.731–0.978)
40%	0.835 (0.655–0.944)
50%	0.772 (0.583–0.905)

**Table 3:** Confusion matrices at different thresholds.

Threshold	Group	Positive	Negative
9.1%	CTEPH	16	0
	Control	4	10
20.6%	CTEPH	12	4
	Control	0	14

**Table 4:** Correlation between perfusion defect fraction and mPAP in the CTEPH Patients.

Threshold	r_S_	95% CI	*p* Value
10%	0.61	(0.16–0.85)	0.0129 *
20%	0.69	(0.30–0.89)	0.0029 *
30%	0.58	(0.11–0.83)	0.0195 *
40%	0.42	(−0.09–0.76)	0.1039
50%	0.33	(−0.20–0.71)	0.2183

Abbreviations: r_S_, Spearman’s correlation coefficient; CI, confidence interval. * indicates *p* < 0.05.

## Data Availability

The datasets generated and/or analyzed during the current study are not publicly available due to restrictions regarding patient privacy and ethical considerations imposed by the Institutional Review Board. However, the numerical data supporting the findings of this study are available from the corresponding author upon reasonable request.

## Supplementary Materials

The following supporting information can be downloaded at: https://www.mdpi.com/article/10.3390/diagnostics16030413/s1, Figure S1: Subgroup analysis stratified by scanner systems; Table S1: Findings of pulmonary angiography of patients in the CTEPH group with similar perfusion defect fraction as those in the control group.
