# Supplementary material for: Diagnostic Performance of Quantitative Lung Perfusion SPECT/CT for Chronic Thromboembolic Pulmonary Hypertension: A Pilot Study
Source: Diagnostics (Basel). 2026 Jan 29;16(3):413. doi: 10.3390/diagnostics16030413 (PMC12897209; doi:10.3390/diagnostics16030413)
Supplement: Supplementary file 1 [file diagnostics-16-00413-s001.zip › diagnostics-4121776-supplementary.pdf]

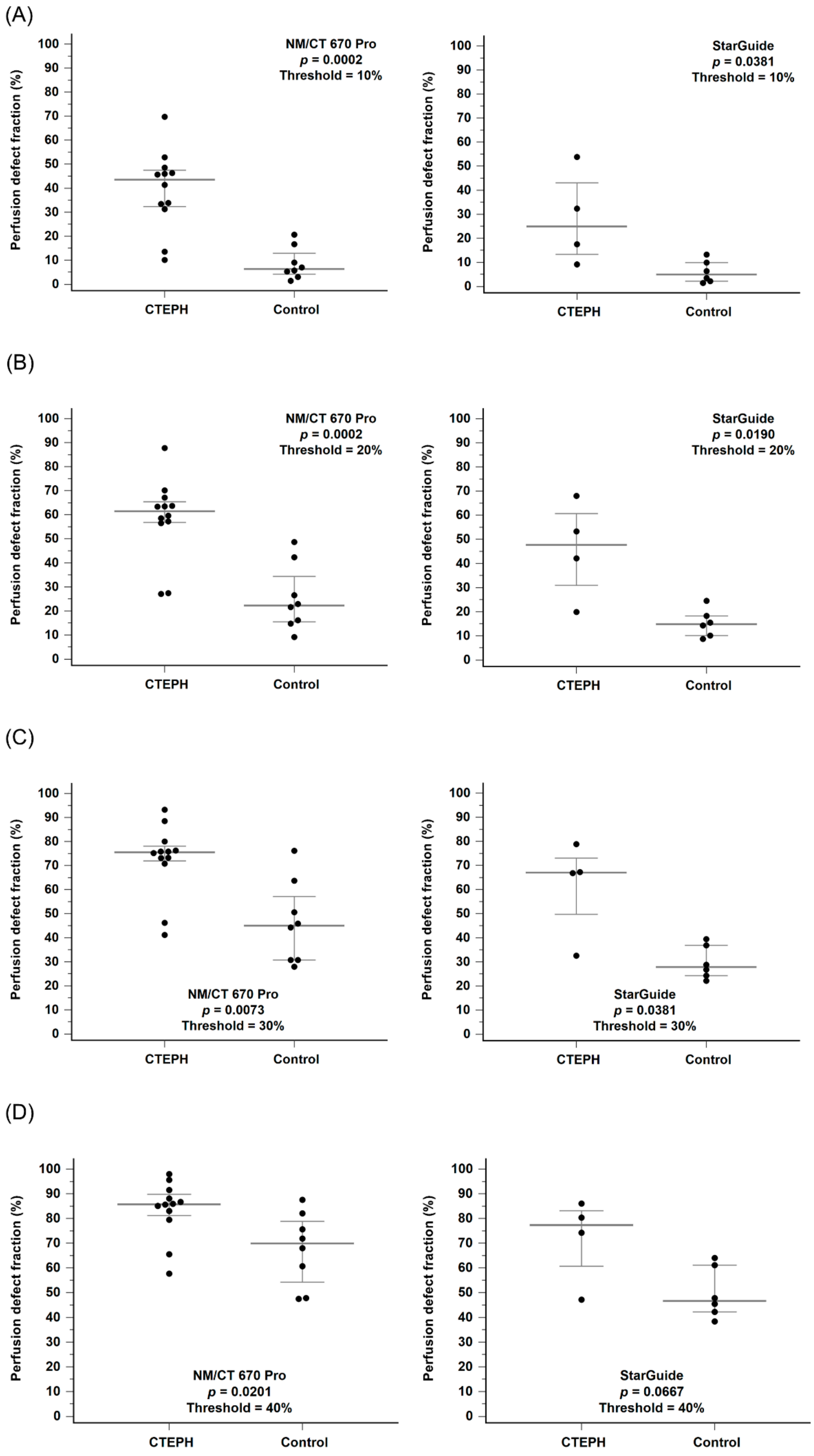
(A)
NM/CT 670 Pro
p = 0.0002
Threshold = 10%
StarGuide
p = 0.0381
Threshold = 10%
(B)
NM/CT 670 Pro
p = 0.0002
Threshold = 20%
StarGuide
p = 0.0190
Threshold = 20%
(C)
NM/CT 670 Pro
p = 0.0073
Threshold = 30%
StarGuide
p = 0.0381
Threshold = 30%
(D)
NM/CT 670 Pro
p = 0.0201
Threshold = 40%
StarGuide
p = 0.0667
Threshold = 40%
Perfusion defect fraction (%)
CTEPH
Control
0
10
20
30
40
50
60
70
80
90
100

S**upplementary Figure 1.** Subgroup analysis stratified by scanner systems. Box plots with scattered dots illustrating the perfusion defect fraction in the CTEPH and control groups under different scanner systems using thresholds of 10%, 20%, 30% and 40% of the maximum perfusion counts, respectively (A–D; left: Discovery NM/CT 670 Pro; right: StarGuide). Statistically significant differences were observed in all comparisons (A–C, $p < 0.05$), with the exception of the StarGuide system at the 40% threshold (D, $p = 0.07$). CTEPH indicates chronic thromboembolic pulmonary hypertension. Long horizontal lines denote median values, and short horizontal lines represent the interquartile range.

S**upplementary Table 1.** Findings of pulmonary angiography of patients in the CTEPH group with similar perfusion defect fraction as those in the control group.

| **Case** | **Findings of pulmonary angiography** |
|---|---|
| Case #1 | Right A1–A2: very distal poor contrast enhancement |
| | Right A7–A10: distal poor contrast enhancement |
| | Left A7–A10: distal no contrast enhancement |
| Case #2 | Right A1–A5: linear lesions |
| | Right A6: ring like lesions |
| | Right A9–10: web lesions |
| | Left A1–6: linear and web lesions |
| | Left A5: web lesions |
| | Left A10: branch total occlusion |
| Case #3 | Right A1–A2: very distal poor contrast enhancement |
| | Right A7–A10: distal poor contrast enhancement |
| | Left A7–A10: distal no contrast enhancement |
| Case #4 | Right A3: poor distal perfusion |
| | Left A1–A2: poor distal perfusion |
